# Supplementary material for: Are carbon emissions trading and green financial instruments synergistic? -Comprehensive quantitative research based on content analysis
Source: PLoS One. 2024 Mar 7;19(3):e0298601. doi: 10.1371/journal.pone.0298601 (PMC10919601; doi:10.1371/journal.pone.0298601)
Supplement: S1 Data — (ZIP) [file pone.0298601.s001.zip › Data/地级市Z_F工作报告.zip!/╣π╢1⁄2/╘╞╕í╩╨/└┤╫╘╣1⁄2╓┌║┼í╛┬φ┐╦╩2╛▌═°í┐_2005.docx]

各位代表：

我代表市人民政府向大会作政府工作报告，请予审议，并请政协委员和其他列席人员提出意见。

2004年工作回顾

2004年是新一届市政府开局之年。一年来，市政府在省委、省政府和市委的正确领导下，在市人民代表大会及其常委会和市政协的监督支持下，努力实践“三个代表”重要思想，树立和落实科学发展观，围绕建设山区经济强市的总目标，坚持以经济建设为中心，抓好政府各项工作的落实。经过全市上下共同努力，取得了经济建设和社会发展的新成绩，圆满地完成了市三届人大一次会议确定的任务，提前一年实现了“十五”计划提出的经济和社会发展目标。

一、国民经济保持良好发展势头

全市完成生产总值257.6亿元，比2003年增长13.7%，增幅提高0.6个百分点，是建市以来发展最快的一年。其中第一产业76.3亿元，增长4.6%；第二产业104.2亿元，增长21.1%；第三产业77.1亿元，增长13.7 %。实现了一、二、三产业协调发展，三大产业的比重由上年31.2：37.9：30.9，变为29.6：40.5：29.9，第二产业主要是工业的比重明显增加。

——农业和农村经济平稳发展。我市农村出现了农林牧副渔五业俱兴的良好局面。全市有效防控了高致病性禽流感疫情，积极组织持续抗旱，实现了农业增产增收。农业总产值111.1亿元，增长5%。粮食播种面积和总产量均完成了省下达的任务，经济作物大面积丰收，养殖业和冬种作物有较大发展。农业产业化经营成效显著，72家龙头企业和13个农民专业合作经济组织带动17.7万农户发展生产，占全市农户总数的31%，户均增收4100元。成功举办了首届农业产业化成果展示会，签约项目投资额7.1亿元。完成造林作业面积24.2万亩，森林覆盖率达到61.1%，我市被评为“广东省林业工作先进集体”。

——工业发展提速，运行质量提高。全市实现工业总产值384.2亿元，增长21.3%。其中规模以上工业总产值227.5亿元，增长20.2%，增幅比上年提高1.7个百分点;销售产值(现价)221.4亿元，增长25%；入库税金6.9亿元、实现利润4.2亿元,增幅分别提高22.8和4.8个百分点；经济效益综合指数125.1 %，提高4个百分点。工业对经济增长的贡献率为46.7%，提高6.8个百分点,拉动GDP增长6.4个百分点。项目建设势头较好，全年共签约投资500万元以上项目133个，签约总额175.2亿元；2003年以来动工（含技改）的99个投资1000万元以上的生产性项目，已有72个投产。

——第三产业有较大发展。全市社会消费品零售总额70.4亿元，增长16%。居民消费价格总指数101.9%，上升1.9个百分点。商贸、服务和房地产业发展较快，通信、信息和中介业等持续发展。开发商品房建筑面积增长25%，销售面积和收入分别增长23%和55.6%；移动、电信、联通等通讯营运商投入大量资金加强设施建设，网络质量和覆盖率不断提高，固定电话（含小灵通）、移动电话用户分别达到45万和53万户，邮政完成业务收入增长12.5%。旅游业逐步升温，全年接待旅客173.7万人次，增长13%。

——财税收入保持较高增长，金融形势稳定。全市地方财政一般预算收入7.2亿元，增长26.3%；支出19.99亿元，增长15.7%。税收入库15.1亿元，增长28.9%。本外币存款余额年末比年初增长10.5%，其中储蓄存款增长12%；本外币贷款余额增长4.3%。多家商业保险机构进入我市，保险市场的竞争格局已经形成。追收地方金融资产2062.4万元。资本市场的开发和利用有了实质性的进展。

——固定资产投资保持较快和合理增长。全市完成全社会固定资产投资76.1亿元(已剔除省投资我市高速公路7.9亿元)，增长28.6%。其中基建增长14.5%，更新改造增长66.4%，房地产增长41.3%。除基础设施和公益性项目外，政府已不再投资竞争性领域，生产性项目投资全部是民资和外资。

二、区域经济实现新发展

——市级经济实力逐步增强。市级税收入库3.2亿元，占全市的21%，增长29%；财政一般预算收入2.3亿元，占全市的31.4%，增长36.2%。省属企业规模扩大，效益提高，转制后的市属企业大部分都得到较快发展。

——县域经济发展加快。五个县（市、区）工业增幅均达17%以上。石材、水泥、不锈钢制品、电线电缆、机械制造、纺织服装等传统行业继续发展壮大，电子、生物制药等新型工业开始兴起；工业园区建设加快，有一批投资超亿元的大项目在建或投产，给县域经济注入了新的活力。各地财政综合增长率较理想，罗定、新兴和云安可获省财政激励机制奖励。

——镇域经济呈现良好势头。镇（街）抓发展的积极性已初步调动，各镇尤其是中心镇加强了工业园区的规划建设和招商引资工作。全市镇（街）共引进500万元以上的项目74个，投资总额20.8亿元，其中已动工54个，投资总额15.1亿元。

三、对外开放和制度创新迈开新步伐

——涉外经济规模逐步扩大。全市新批准外商直接投资企业59家，合同外资金额11411万美元，增长13.2%；实际吸收外商直接投资（老口径）5389万美元，增长27.9%。进出口总额60234万美元，增长23.5 %。其中出口总额34959万美元，增长22.9%；一般贸易出口8079万美元，增长45.6%；加工贸易出口26880万美元，增长17.4%。我市与挪威王国拉尔维克市签署了关于友好合作关系的共识备忘录；举办了首届海外云浮乡亲联谊大会，港澳同胞、台湾同胞和海外侨胞纷纷到我市考察，使我市对外交流进一步加强。

——东渐西联取得良好开端。我市与贵州省黔西南州缔结为友好市州，有利于优势互补，共同加快发展。先后参加了首届泛珠三角区域经贸合作洽谈会、粤港台经济技术贸易合作交流会和第三届“山洽会”，共签订项目84个，合同金额86.2亿元和1.5亿美元。从珠三角地区转移到我市发展的企业，数量逐步增多。

——民营经济快速发展。全市新登记个体工商户7006户，增至39632户；私营企业增加327家，增至1294家。拥有自营进出口权的民营企业增加13家，达到36家。民营企业缴纳税金7.8亿元，占全市税收51.7%。一批基础较好、实力较强的民营企业规模继续扩大，其中温氏集团和亨达利集团分别列全省百强民营企业的第8位和第19位。民营经济已成为我市最活跃的经济增长点。

——国有资产管理体制和国有企业改革加快。组建了市国有资产监督管理机构，国有资产营运和监管工作进一步规范。国有企业改革继续推进，改制率达94.1%。国有企业资产有效盘活，人员、债务得到妥善处理。去年，市财政用于市直职工安置费用达1141万元。

——其他各项改革稳妥推进。农业税税率减低50%，农民负担进一步降低。深化财政管理体制改革，在全额拨款单位推行部门预算，其中市直已全部实施，并统一了公务员岗位津贴，在10个单位开展了国库集中收付改革试点工作。积极推进住房公积金制度建设，保证公积金高效运行，当年公积金归集总额增长91.6%，住房贷款增长42.3%。完成了市、县政府机构改革和撤村并镇工作。人事、文化、广播电视、卫生等体制改革顺利推进。

——整顿和规范市场经济秩序取得新成效。严厉打击制假售假、走私、传销等违法活动，加强食品、药品安全监督和管理，强化了特种设备的安全监察。查处各类经济违规违法案件1875宗，其中不正当竞争案件86宗，制售假冒伪劣案件326宗，无照经营案件911宗，涉农案件111宗。按省的统一部署，对车辆超限超载运输进行了治理。

四、投资硬环境进一步改善

——城镇建设步伐加快。编制完成了《云浮市市域城镇体系规划（2004—2020）》，各县（市）城区和中心镇的规划修编加紧进行。市区总投资17亿元的18个主要城建项目，已动工15个，投入资金2.6亿元；对城区主要出入口进行了改造，市电信综合大楼和群众艺术馆等投入使用，市档案馆、土地交易中心、妇儿活动中心、金山大道、街道立面装饰以及住宅小区等建设进展顺利，市区绿化、美化工程继续推进，启动了南山森林公园和生态公益林建设工程。各县（市）城区建设力度加大，城镇基础设施不断完善，辐射和集聚功能逐步增强。村镇建设加快，全市共投入村镇建设资金6.3亿元，增长12.6%。其中投入中心镇建设4.5亿元，完成城建项目61个。建成生态示范村113条，受惠农民5.6万人。

——交通建设取得新突破。全市共投入公路建设资金15.1亿元，改造、建设等级公路和镇村公路865.5公里，分别增长42.3%和86.5%。高速公路马安至河口段建成通车，云城河口至郁南平台段以及罗定支线抓紧筹建，罗定至岑溪粤境段预可行性报告通过评审；国道324线河口至城区段改造工程已动工，省道113线新兴县城至阳春段、352线罗定至信宜段基本建成；改造了一批客货运站场,客货运周转量增加。西江云浮段“文明样板航道”通过国家验收，沿西江港口总体规划通过专家评审，部分港区建设项目正在进行。全市港口货物吞吐量542.8万吨，增长10.4%。

——电力和电网建设扎实推进。全年总发电量56.4亿千瓦时,增长33.2%；全社会总用电量20.3亿千瓦时,增长17.8%。罗定粤泷发电厂建成并网发电，云浮电厂三期、华润云浮电厂等项目正按程序申报立项。农网和城网改造建设进展顺利。

——环保、水利等设施建设加快。西江水环境综合治理工程动工项目30个，已完成5个。石材废渣、水泥粉尘等污染源得到有效治理，市区每平方公里月降尘量降至7.4吨，连续两年达到省的标准。市城区医疗垃圾处理站投入使用，生活污水处理厂即将竣工，生活垃圾填埋场开始兴建；各县城的环保设施正在抓紧建设。落实了13宗城乡水利防灾减灾工程项目，29宗小型水库除险加固实现了达标。

五、群众生产生活条件不断改善，各项社会事业取得新进展

——城乡居民生活水平有较大提高。城镇在岗职工工资总额17.6亿元,增长19%；农民人均纯收入4003元，增长5.4%；全市城镇人均居住面积超过22平方米，居全省较前位置。

——群众关心的热点、难点问题得到逐步解决。开展了农村征地专项执法监察工作，清理兑现拖欠的征地补偿款10899万元, 为农民工追回工资5278万元，全面完成了清欠任务;清理建设领域历年拖欠工程款工作也取得了较好成效。加大了解决“四难”和其他热点、难点问题的力度。全年共办理法律援助案件685件；为23082名贫困学生减免了书杂费；扶助583户农村贫困户和67户城镇特困户解决了住房难问题；投资1007万元，为5.8万农民解决了饮水难问题;投入3060万元，帮助18个水库移民村、673户水库移民解决了住房、行路难等问题。加大了对贫困村的帮扶力度，年纯收入超过3万元的村增加59个，全市村干部人均月报酬已达到或超过300元。

——社会保障体系不断完善。社会保险实现了市级统筹。全市参加城镇职工基本医疗和企业养老、失业保险人数分别比上年底增长8.0%、7.1%和6.4%；社保费征收总额3.5亿元，增长20 %，实现了当期收支平衡；新增就业岗位3.4万个，下岗失业人员再就业9910人；城镇登记失业率为3.3%，低于省下达的控制指标；加大了农村劳动力培训力度，农村劳务经济进一步发展；市、县财政继续拨款免费培训城乡贫困人员和下岗失业人员。完成了2005年度农村合作医疗和补充医疗保险的发动缴费工作，覆盖率扩大至82.9%和66.8 %。农村特困户和五保户全部纳入合作医疗，所缴费用全部在医疗救助金支付。优抚政策得到进一步落实，企业军转干部的工资待遇得到较好解决。全市纳入“低保”人数占全市总人口的14.6‰，比上年提高2.4个千分点。发放救灾款357万元，帮助受灾群众近5.6万人次。农村五保工作得到落实，五保供养经费全部由财政解决。新建了一批社区康复服务指导站点，精神病人和脑瘫、聋哑、弱智儿童的救助、康复工作得到加强。

——科技、教育、文化、卫生、体育、人口和计划生育等工作有较大进步。科技创新综合能力和地区竞争力得到提升。新增省级科技进步载体33个，有9家企业被评为全国优秀科技企业；新增1个国家免检产品、4个省名牌产品、4个省著名商标；科技进步对经济增长贡献率达到47%，高新科技工业产值比重达到15%，分别比上年提高1个和6个百分点。教育事业进一步发展。各级各类教育办学规模不断扩大，“双高”普九义务教育稳步推进，普通高中教育有新发展，老区山区学校改造、学校布局调整和等级示范学校建设取得较好成绩。“一机两语”教育走在全省山区市前列，全市完全小学已全部从一年级起开设英语课。教育质量提高，高考再创好成绩。文化、广播电视设施增加，文化生活进一步丰富。全面启动了《云浮市志》修编工作。卫生医疗服务水平不断提高，有效地防控了非典等各种疫情，农村中医工作被列为全省两个创建先进示范市之一。体育事业发展加快，举办了第五届“体育节”，全民健身运动蓬勃发展，体育竞技水平不断提高。全市人口和计划生育工作全面达标，人口自然增长率5.71‰，完成了省下达的年度人口控制计划。全市完成基本农田保护任务169.4万亩,新开垦耕地5019亩,实现了年度耕地占补平衡；治理整顿土地市场秩序取得预期效果，地质灾害得到有效防治。开展了第一次经济普查和“十一五”规划编制工作。发展改革、物价、信息产业、知识产权、经济发展研究、民族宗教、档案、人防、武装、国防动员、民兵预备役、外事侨务、台务、消防、保密、新闻出版、文联、社科联、妇女儿童、红十字会、气象以及驻外机构等也取得了新的成绩。

六、民主法制建设深入推进，社会大局保持稳定

——民主渠道进一步拓宽。坚持和完善向人大及其常委会报告工作、向政协通报情况制度，自觉接受监督，人大及其常委会的决议得到认真执行，重大问题广泛征求市人大代表、市政协委员意见。全年共办理市人大代表建议81件，市政协委员提案62件，市三届人大和三届政协一次会议的代表建议、政协委员提案已全部办结。加强与工会、共青团、妇联、工商联、科协等人民团体的联系，认真听取人民群众对政府工作的意见和建议。健全信访工作网络，落实领导信访包案责任制，群众信访反映的突出问题绝大部分得到解决。基层民主建设加强，政务、厂务、校务、村务公开和农村居民自治、企业职工代表大会等制度进一步完善。

——政府依法行政能力逐步加强。认真贯彻落实《行政许可法》和《全面推进依法行政实施纲要》，进一步完善行政复议制度，政府机关法律意识增强，依法行政水平逐步提高。市政府全年制定实施规范性文件13件，废止规范性文件14件，取消不符合规定的行政许可93项，政府工作进一步规范化、法制化。市直单位实现了电子公文交换，提高了办事效率。行政服务中心的办事制度更为完善。在全市财政、计划生育、民政系统和部分镇（街）、社区及电信等行业开展民主评议行风工作，机关作风逐步好转。设立了市民营企业投诉中心，较好地维护了投资者的合法权益。加大了对重点领域、重点部门、重点资金的审计监督。有形建筑市场和土地市场健康发展，工程招标率和工程公开招标率均为100%，经营性用地和采矿权有偿出让全部实行公开交易。政府采购实现了采购与监督职能相分离，招标采购进一步规范。县级以上公立医疗机构全部进行药品和医疗器械集中招标采购。继续加大对公路“三乱”和其它乱收费、乱罚款的治理力度，加强中小学“一费制”管理，各类乱收费、乱罚款现象得到有效遏制。

——社会治安综合治理成效显著。认真组织打击“双抢”、“双盗”和侦破命案专项行动，恶性案件大幅度下降。全市共侦破各类刑事案件2012宗，命案侦破率位于全省前列。大力打击“六合彩”等赌博活动，共查处“六合彩”赌博案件600多宗，抓获庄家、赌头215人。坚决打击和取缔“法轮功”等邪教组织，维护了国家安全和政治稳定。深入开展人民调解工作，积极调处民间纠纷，全年没有发生重特大群体性事件，全市社会稳定。

——安全生产形势稳中趋好。认真落实安全生产责任制，抓好安全生产专项整治，各类事故起数、死亡人数、直接经济损失同比下降，全年没有发生重特大安全生产事故。

各位代表，去年我市经济和社会发展取得的成绩，是上级党委和政府的正确领导，全市人民和社会各界人士奋力拼搏的结果。在此，我代表市人民政府，向全市广大工人、农民、知识分子、干部、驻云浮解放军、武警官兵和公安干警以及其他各界人士，表示崇高的敬意！向关心和支持云浮建设的香港、澳门同胞，台湾同胞，海外侨胞和国际友人，表示衷心的感谢！

去年我市经济和社会事业虽然取得比较明显的成绩，但仍存在不少薄弱环节。主要有：一是二、三产业比重偏低，尤其是工业总量小，工业增长速度落后于全省平均水平。二是经济结构不合理，资源、能源消耗大的企业比重大，高新技术企业少，容易受原材料和能源价格波动影响。三是农民增收缓慢。四是财力较弱，财税征管工作有待加强，财税占GDP比重较低。对这些薄弱环节，我们将在今后的工作中，采取切实有效措施，逐步加强。

2005年工作总体要求和目标任务

各位代表， 2005年是我市贯彻落实市第三次党代会精神，建设山区经济强市，构建和谐云浮的重要一年。当前，我们正面临经济发展的重要战略机遇期，扩大内需的累积效应和巨大市场潜力将进一步释放，泛珠三角区域合作与发展、珠三角等经济发达地区产业转移和产业扩张趋势将继续增强；我市基础设施和发展经济环境日益改善，经济状况逐年好转，各级政府领导经济发展的能力不断增强，广大干部群众要求加快发展的愿望强烈。所有这些，都表明我市基本具备了加快发展的条件。只要我们解放思想，创新观念，把握好新的发展机遇，加快推进改革和经济结构调整，用改革的办法破除体制上的障碍，用创新的思路破解发展中的难题，用求真务实的精神抓好工作的落实，就一定能够实现全市经济和社会的快速、协调、健康发展。

2005年政府工作的总体要求是：高举邓小平理论和“三个代表”重要思想伟大旗帜，进一步树立和落实科学发展观，认真贯彻中央经济工作会议、省委九届六次全会以及张德江同志视察云浮重要讲话精神，围绕建设山区经济强市、构建和谐云浮总目标，积极实施“东渐西联、优内拓外、错位竞争、统筹并进”发展战略；坚持突出发展工业，以工业化带动城镇化、农业产业化，加快市、县、镇三级经济发展；坚持大力推进科技、教育、文化、卫生等各项事业，使全市社会事业与经济建设相辅相成，相互促进，协调发展；坚持依法治市、依法行政，加强政治文明和精神文明建设；坚持以人为本，注重社会公平，加强利益协调，构建和谐社会。通过扎实的工作，促进国民经济持续快速协调健康发展和社会全面进步，为实现山区经济强市和全面小康社会宏伟目标打下坚实基础。

全市经济和社会发展的预期目标是：生产总值增长13.5%，工业增加值增长22%，社会消费品零售总额增长15 %，固定资产投资增长20%，地方财政一般预算收入增长15%，外贸出口总值增长15%，实际利用外资增长25%，农村居民人均纯收入增长5%，人口自然增长率7‰，城镇登记失业率3.4%，城镇居民人均可支配收入增长12%，居民消费价格总指数增长3%。

为实现上述总体要求和预期目标，我们必须坚持走工业化、城镇化和农业产业化路子，市、县、镇经济一齐抓，一、二、三产业一齐上，创新工作抓落实机制，充分调动各方面积极性，努力使全市经济实现加快发展。

一、加快工业发展速度，提高工业化水平

我市工业量少质弱，粗放经营，整体水平低,制约了全市经济的快速发展。我们要进一步强化加快发展工业的意识，把它作为经济工作的重中之重，加大项目建设力度，不断优化结构，走追求质量和效益的新型工业化之路，努力提高全市工业化水平。

提升现有工业的整体水平。做大做强现有工业企业，既是做大全市经济总量的重要基础，也是优化我市工业结构、提高工业化水平的重要工作。各地要帮助现有企业挖掘生产潜力，千方百计组织满负荷生产。同时，积极引进外资和运用高新技术改造提升传统工业，支持支柱行业和重点企业增资扩产、技术改造，提高工艺技术、产品档次和管理水平，增加经济效益；引导有一定基础的企业实施品牌战略，帮助企业借品牌、创品牌、出名牌，争创全省同行业一流企业。

大力推进项目建设。建立和健全项目工作责任制，对重点项目实行责任到人抓落实的办法，确保项目建设的落实。切实帮助在建项目，尤其是规模比较大的水泥生产项目，以及生物制药、电子、新材料等高技术含量、高附加值的项目加快建设进度，尽快形成生产能力，早出效益。继续加大项目的引进力度，重点建设一批投入和产出大、经济拉动力强、对财政贡献大的骨干项目；积极扶持高新技术产业，大力发展低能耗、低物耗、低污染、少占地的集约型企业，以此带动配套产业和中小企业群发展；加强已签约项目的跟踪落实；抓好云浮电厂三期、华润云浮电厂、罗定粤泷电厂二期等重大项目的申报立项工作。同时，抓紧开展郁南向阳抽水蓄能电站的可行性论证工作。

积极发展园区经济。坚持高起点规划、高标准建设、高效能管理的原则，多方筹措资金，加快工业园区建设。利用现有工业园区，加快资源整合和功能配套，促进项目、资金、技术、人才向园区集中；抓紧规划和建设都杨工业园、市高新技术产业园、新兴水台生态工业园等重点园区，使其成为我市经济发展的新亮点；抓住土地利用总体规划修编的机遇，充分利用中心镇的优惠政策，采取投资多元化、合作多样化的办法，抓紧建设一批县、镇工业载体。今后，工业项目原则上进园区，严格控制项目单独征地，逐步推行不同地区不同行业单位土地最低投资和产出标准管理，限制占地多、投入少、效益差的项目，提高用地的集约度，满足工业发展的用地需求。

加快几大工业板块的协调发展。市属企业和省管企业、县属企业、镇域企业，是我市工业的三大板块。壮大全市经济总量，必须实现三大板块协调发展。市属工业企业多年来虽然有一定发展，但仍然相当薄弱。采取切实措施，加快市属工业发展，壮大市级经济实力，是加快经济发展的一项硬任务。要下大力气盘活现有企业资产，使老企业焕发生机；破解市属工业发展空间不大的难题，实行市、区联办工业载体和市、县利益共享、联合办企业。同时，积极支持云硫集团、云浮电厂等省管企业拉长产业链或跨行业发展，扩大经营规模。县属企业虽比市属企业强一些，但仍然堆头太小，同样要加快发展步伐。镇域工业是又一薄弱环节，要制订扶持政策和激励措施，调动镇（街）发展工业的积极性，加快镇域工业的发展，创建一批工业镇，增强镇级经济实力。

加强对工业经济的服务工作。落实好各级鼓励加快工业发展的优惠政策，支持企业做大做强；加强经济运行调控，协调解决企业用电、用煤、用油、用地和招工难等实际问题；各金融机构要运用好国家的金融政策，加强和完善金融服务，搞好银企合作，全力支持工业经济的发展；加快资本市场的开发，成立担保公司，帮助中小企业解决融资难问题；积极协助亨达利水泥制品有限公司和温氏集团等企业做好上市前期工作，同时支持符合条件的企业通过资产重组，组建股份制公司，为做大做强打下基础；加强与高等院校和科研院所的全面合作，为工业发展提供科技动力和智力支持。

二、以农业产业化为龙头，全面推进农村经济发展

近年来，我市解决“三农”问题力度逐年加大，但粮食增产和农民增收的基础仍不稳固，农民增收的水平提高不快，农业基础薄弱的状况还没有明显改变。各级政府要正确分析农村形势，切实重视和解决“三农”问题。认真贯彻落实2005年中央1号文和省委、省政府《关于统筹城乡发展加快农村三化建设的决定》，加大工业反哺农业、城市支持农村的力度，提高农业综合生产能力。加快农村经济发展，增加农民收入。认真落实粮食工作政府首长责任制和种粮大户直接补助政策，加强对基本农田的保护和建设，确保完成粮食生产、储备及风险基金任务，加强粮食市场监管，确保全市粮食安全。与此同时，坚持以农业产业化为龙头，带动农村经济和社会全面发展。

进一步壮大龙头企业，提高农业产业化水平。运用现代工业的经营理念谋划发展农业，大力推广“公司+农户”或“公司+基地+农户”的经营模式。支持和帮助现有农业龙头企业解决资金、技术和管理等问题，实施名牌战略，促其健康成长，增强带动能力；重视引导和培育以种养业、农产品加工以及农产品流通为主营业务的龙头企业的发展，引导有实力的龙头企业带动石灰岩苦旱镇和山区镇农民发展养殖业；动员和运用社会资金建设一批农林产品批发市场，逐步推进网上交易等现代信息技术，进一步拓宽农林产品物流平台；鼓励农林产品深加工，延长产业链条，增加附加值。

积极发展特色农业，提高农业经济效益。我市的特色农业已有一定基础，全市已建成了优质稻、蔬菜、水果、肉桂、茶叶、蚕桑、竹子、松脂、禽畜、花卉等十大农业商品基地，其中有部分产品已成为全国品牌。各地要在原有基础上，结合实际，发挥优势，瞄准市场，以建设全国知名的农业种养加工基地为目标，继续优化特色农业的产品结构，引导大宗农产品向优势产区集中，扩大优质、高产、高效、生态、安全的特色农业规模，促使农业经济提高到一个新的水平。

大力改善农村生产生活条件。继续抓好村道硬底化建设，改善农村交通条件。搞好通讯设施的规划和建设，扭转农村尤其是山区农村通讯落后状况。加强水利设施的建设、维修和管理，推进水利抗旱治本和机电排灌工程建设，提高农田灌溉能力。办好标准化农业生产基地和产业化示范区，引导农民运用标准化技术发展农业生产。加强农业生产资料市场管理。建立完善动植物疫情报告、预警机制和农产品质量安全管理体系。切实搞好农村信用社改革试点工作，增强农信社支持和服务“三农”的能力。

深化农村各项改革，切实减轻农民负担。全面落实免征农业税政策，落实减轻农民负担行政首长负责制和部门责任制，大力治理涉农乱收费。加快农村集体土地所有权登记发证、农地分等定级和土地更新调查工作，完成换发林权证任务。加强农村集体征地补偿款分配和使用的监督管理，坚决查处侵犯农民合法权益的违法行为。搞好村级换届工作，完善村务公开和民主管理形式，规范集体资产和财务管理。

三、大力发展县域经济，壮大全市经济总量

县域经济在我市国民经济中占有重要的地位，强市之基在于强县。各地要坚持以工业化为核心，以城镇化建设为依托，以农业产业化为基础，以园区为载体，以科技和制度创新为动力，以招商引资为突破口，以大力发展民营经济为重要途径，千方百计发展壮大县域经济。

着力培育优势行业。各县（市、区）应从比较优势出发，制定本地区工业和产业发展战略，选准行业发展方向，形成有县域特色的工业结构。依靠科技进步增强传统企业和产品竞争力，做大做强水泥、石材、机电、不锈钢制品、化工、电子、纺织服装和农林产品加工等行业。各县（市、区）应加强行业前景的分析研究，抓住本区域有一定规模和基础的行业，制订扶持措施，将其发展为全省乃至全国市场份额大、知名度高的支柱行业。

大力发展第三产业。我市第三产业发展滞后，制约了全市经济质量的提高。各地应加强第三产业的规划和发展工作。支持金融改革和创新，提高信贷质量，维护正常的金融秩序，促进金融稳定发展；支持商业保险实行公平竞争，积极开发新险种，发展新业务，发挥其对经济和社会发展的促进作用。加快交通营运、商贸和餐饮服务等传统行业发展，加大西江航运的开发力度，培育物流、连锁经营、信息、中介和社区服务等新兴行业。各县城要在搞好规划的前提下，建设一些水准较高的商贸区和专业市场，刺激消费；利用民资和引进外资建设一批档次较高的旅业和餐饮业设施，提高城市服务能力及城市品位。中心镇也要重视发展第三产业。市区、县城和中心镇不准新批地单家独户建私房，要激活二、三级房屋交易市场和开发档次较高的住宅小区，加快房地产业的发展。各地还要搞好旅游规划，整合旅游资源，打造旅游品牌，发展具有地方特色的旅游服务业，扩大旅游经济规模。

继续大力发展民营经济。落实省、市的一系列优惠措施，营造有利于民营经济发展的更为宽松的社会环境。鼓励民资进入基础设施、垄断行业、公用事业以及法律法规没有禁止的其它行业和领域。支持发展科技型、出口创汇型、就业型和农副产品深加工型企业；引导民营企业开展制度创新、技术创新和管理创新，依法经营，创造品牌，促进民营企业做大做强。

加快发展镇域经济。以工业化为重点，同时加快城镇化和农业产业化建设步伐，着力加快中心镇建设，扩大镇域经济总量。一是加大招商引资力度，发展劳动密集型企业，为农村富余劳动力创造更多就业机会；二是做好利用地方资源发展经济这篇文章，积极发展利用矿产、水、农林产品等资源型特色经济；三是继续培育农业龙头企业，打造农业品牌，提高农业经济综合竞争力；四是加强城镇规划建设，完成城镇建设和土地利用的总体规划修编工作。从今年开始，实行镇域经济工作考核制度，调动各级的积极性和创造性，推进镇域经济的发展。

为优化县域经济发展环境，凡省已下放给地级市的管理权，除法律法规另有明确规定外，一律下放给县（市）。

四、加大外经贸工作力度，做大外向型经济

加强对外贸易，提高出口竞争力。巩固港澳、东南亚等传统出口市场，大力开拓欧美、非洲等市场。优化出口商品结构，扩大高新技术产品、机电产品和农产品出口，大力扶持生产规模大、特色强、有国际竞争力的产品出口，提高出口产品附加值；帮助中小企业开拓国外市场。促进加工贸易转型升级。加强口岸建设，搞好涉外服务，抓好出关绿色通道、电子海关等工作，落实出口退税政策，激励涉外企业提高出口积极性。

全方位推进招商引资。积极利用省政府关于山区及东西两翼与珠三角联手推进产业转移的政策，建立珠三角外资企业转移对接平台，积极创造条件迎接珠三角产业转移；整合各地驻珠三角招商联络处资源，形成招商引资合力，力争在珠三角新一轮产业转移中取得实效。创新招商引资方式，推行以商引商、委托招商、小分队招商、网上招商等方式，利用龙头项目带动产业招商。积极鼓励现有大型外资企业增资扩产，同时借助他们的影响力，吸引业务相关或上下游配套企业。加强外商投资服务工作，努力创造工业用地、用水、用电价格优势，降低营商成本，增强投资吸引力。建立健全招商引资工作责任制，调动和发挥各方面招商引资的积极性，加强招商引资的组织、协调、落实工作，实行责任制到人，全程跟踪服务。

五、强化财税工作，增强全市财力

大力培植税源和强化税收征管。重点加快在建项目建设进度，促进新办企业早出效益，帮助现有企业扩产增效，鼓励“三资”企业拓展国内市场。各有关部门要支持和配合税务部门强化税收征管，不断推进税收精细和科学化管理；改进税收监测和征管手段，合理调整中小企业和个体工商户的税负；加大税收稽查力度，严厉打击各种偷、逃、骗税行为，做到应收尽收，确保财政收入稳定增长。税务部门要加强企业财务指导，加大税法的宣传，促进企业如实申报，依法纳税。

加强政府资源开发管理。加强国有资产管理，整合和盘活国有闲置资产，防止国有资产流失。加快政府资源的开发利用，运用市场手段强化对土地、矿产、水等资源以及其他专营性资源的管理和经营，实行有偿使用。

深化财政改革，优化支出结构。积极探索和逐步建立有利于调动各方面积极性，与经济工作绩效挂钩的财政激励机制。认真运用好有限的政府财力，严格控制一般性支出，确保公共事业性经费和重点项目的支出。今年，市直全额拨款单位全面铺开国库集中收付制度改革，各县（市、区）也要开展国库集中收付制度改革试点工作。扩大政府采购领域和规模，进一步健全和严格市直单位统一岗位津贴管理制度。严格和完善非税收入的收支两条线管理，加大检查力度，对截留非税收入或设立单位“小金库”，滥发钱物、公款旅游及铺张浪费的，必须严肃查处。

强化财政资金监管。加大各级财政收支的审计和监管力度，严格政府投资项目的监控，抓好资金分配、流转及使用等环节，完善财政支出绩效评价体系，健全财政系统内部监督机制，从制度上防止截留、挤占、挪用、浪费财政性资金行为。

六、加强基础设施建设，不断改善投资环境

加快城镇建设。搞好城镇规划，促进城镇协调发展。市区要加快市委党校、档案馆、图书馆、博物馆、妇儿活动中心、市中级法院审判大楼、金山大道、南山森林公园等项目建设；促进上等级的旅业、餐饮和商贸项目建设；推进住宅小区开发，促进房地产业发展；继续启动一批美化、绿化工程，进一步改善城市居民的生产生活环境。切实抓好县城建设，突出特色，提高规划水平，加快建设进度。完善中心镇的环境卫生、供水、排污、医疗、教育、商业服务等设施，增强集聚能力。要加强城镇周边的生态公益林建设，提高城市绿化水平。

继续推进交通等基础设施建设。协助和配合省交通部门做好河口至郁南平台及罗定支线高速公路动工建设工作，开展罗定至岑溪、云浮（云城思劳）至新兴、云浮（郁南东坝）至阳江港高速公路前期工作，抓好国道324线河口至城区段及省道369、280、276线改造工程，年内完成县通镇公路改造；推动罗定至广西岑溪铁路建设；加快云浮港的报批立项和建设步伐；继续完善供电、供水和信息网络等设施；抓好市区南山河等11宗城乡水利防灾减灾和61宗小型水库除险加固达标工程。

七、统筹推进科技、教育、文化等各项社会事业发展

提升科技创新能力。建立科技与经济紧密结合的创新机制，加快科技服务体系建设。帮助企业加大资金投入，加快用高新技术改造传统行业和企业，提高产品科技含量；扶持骨干企业组建科研中心，提高企业自主开发能力。进一步加强农业科技推广体系建设，提高农业科技水平。加强与高等院校合作，加快组建华南理工大学云浮研究院；引进科研成果发展高新技术产业，逐步优化我市产业结构。

全面推进教育事业。统筹发展基础教育、职业技术教育和成人教育，规范幼儿教育。抓好示范性高中学校建设，扩大普通高中和职业学校办学规模；加快市技工学校筹建工作；继续加强素质教育，提高“一机两语”特色教育水平；建立教育强镇督导评估制度，启动教育强镇试点工作。

加快发展文化、广播电视、新闻出版事业。加强文化和广播电视设施建设，健全公共文化服务体系，大力开展民间文化、社区文化、企业文化、校园文化活动，丰富人民群众精神生活；挖掘本地历史文化资源，发展特色文化；整顿、规范文化市场；引进培养文化人才；改革文化、广播电视和出版体制，发展文化产业和新闻出版业，促进文化与经济互动发展。

推动人才、卫生、人口和计划生育等事业发展。实施人才强市战略，完善人才选拔任用机制，扩大人才队伍。启动省级卫生城市创建活动，促进疾病预防控制、卫生监督执法和医疗救治三大体系建设，加快创建农村中医先进市步伐。深入开展全民健身活动，抓好市区健身广场、市体育公园等体育场地、设施规划和建设，做好省运会的备战参战工作。全面落实人口和计划生育政策，提高人口素质，综合治理出生人口性别失衡问题。加强国防教育和国防后备力量建设工作，全面落实优抚政策，开展省级双拥模范城市创建活动。落实民族、宗教政策。规范民间组织管理。认真实施妇女儿童发展规划，切实维护妇女儿童合法权益。关心残疾人康复和就业。抓好“十一五”规划、经济普查和人口抽查工作。进一步做好统计、外事侨务、台务、信息、保密、消防、人防、气象、档案、地方志等工作。

八、千方百计增加群众收入，努力提高人民生活水平

解决民生问题，增加群众收入，改善人民生活，是政府工作的根本出发点和最终落脚点。除要通过加快发展增加城乡居民收入外，还要突出抓好几项工作：

加强劳动保障工作。努力增加就业岗位，进一步扩大城乡就业门路；加大劳动监察力度，规范企业用工行为，坚决制止和查处拖欠工资、侵犯劳动者权益等行为，健全劳动关系协调机制，保障劳动者合法权益；落实再就业政策，帮助国有企业下岗人员再就业；充分发挥劳动力市场的作用，加快城乡统筹就业；继续加大智力扶贫力度，各级财政要安排专门预算，整合政府办学资源，启动社会力量办学，扩大农民技能培训规模，促进农村剩余劳动力向城镇转移和外出就业，推进农村劳务经济的发展。

加快城乡社会保障体系建设。完善城乡最低生活保障和农村五保供养制度，建立城乡特殊困难群众的社会救助体系。开展社会保险地税全责征收工作，继续扩大社保覆盖面，加强保费征收和管理，确保养老基金当期收支平衡，为逐步做实保费个人账户创造条件；逐步解决企业早期退休人员养老金偏低问题。进一步完善新型农村合作医疗管理制度，努力从根本上解决农村“治病难”问题。

积极解决困难群体实际问题。大力推进“一保五难”工作。继续实施农村饮水解困和水库移民村住房改造工程，年内解决18万农民饮水难问题，帮助16条水库移民村解决住房、行路难问题；进一步加大工程款的清欠力度；落实扶助措施，解决贫困家庭子女入学问题；继续实施农村特困户危房改造计划；做好农村低收入群体的动态监测工作，有针对性地进行扶持和帮助。

九、坚持以人为本，建设和谐云浮

推进社会主义思想道德建设。加强社会公德、职业道德和家庭美德教育，规范市民行为，提高市民素质。在全市开展“爱国、守法、诚信、知礼”教育，建立和谐的人际关系。倡导科学、文明、健康的生活方式，建设节约型社会，深入开展文明城市、文明镇村、文明行业、文明单位、文明社区和军警民共建等创建活动，进一步提高公民的文明水平。加强信用道德建设，加快建立面向个人和企业、覆盖经济和社会各个方面的信用体系。加强科普宣传，倡导科学精神。

加强社会治安综合治理，维护社会稳定。严密防范、打击 “法轮功”等邪教组织的非法活动。始终保持对犯罪分子的高压态势，严厉打击各种刑事犯罪，重点打击“双抢”、“双盗”等多发性侵财犯罪、有组织犯罪、毒品犯罪以及严重暴力犯罪。积极探索新形势下加强治安管理的新路子，深入推进社区警务和农村治保会、调解会建设，加强对娱乐服务场所、特种行业、出租屋、非户籍人员管理，做好城市生活无着的流浪乞讨人员的救助管理和刑释解教人员安置帮教工作。

加强环境监督治理和生态屏障建设。重点抓好西江、新兴江、南山河、南江河综合整治，加快城市生活污水、粪便和垃圾处理设施建设，加强水泥等企业的污染物排放在线监控，落实石材加工企业循环用水监控制度和废渣处理措施，整治污染源；严格执行环境保护前置审批、环境影响评价和“三同时”制度；狠抓城市环境治理，加强对道路遗撒、工地扬尘和噪声管理；推动发展循环经济；推进林业生态县建设，加快生态公益林体系和林业重点工程建设，合理调控生态公益林与经济林的比例；强化林地、林木、野生动植物保护，合理开发和节约使用自然资源，保护土地和水资源。依法查处无序开发资源等严重破坏生态的行为。继续规划和建设第二批生态示范村。

做好安全工作。落实社会单位主体责任和政府监管责任，积极推进安全质量标准化，规范安全生产行为，从源头上防范各类安全事故；加大检查监测力度，确保农副产品、食品、药品、保健品、化妆品等安全；完善应急体系，增强反应和处置能力。

做好信访工作。进一步完善领导信访接待和包案责任制度，积极化解各类社会矛盾，继续做好集中处理信访突出问题和防范群体性突发事件；加强基层调解工作，完善人民调解、治安行政调解、司法调解相衔接的工作制度；努力解决信访积案。

十、切实加强政府自身建设

面对我市建设山区经济强市与创建和谐云浮的新形势、新任务、新要求，政府各部门必须牢固树立科学发展观和正确的政绩观，按照科学执政、民主执政、依法执政的要求，推进社会领域的各项改革，加快政府职能转变，在搞好经济调节和市场监管的同时，更加重视发挥社会管理和公共服务职能，不断提高行政效能和服务水平。

全面推进依法行政，建设法治政府。认真贯彻《行政许可法》，加快行政审批制度改革。更新行政理念，完善行政程序，规范行政行为，做到依法行政、程序合法、高效便民、诚实守信、权责统一。积极完善政务公开、收费公示制度，加快推行电子政务，进一步提高行政效率，优化政务环境。

转变机关作风，狠抓落实。加快我市经济和社会发展，需要各级政府及其部门全体工作人员增强责任感和事业心，忠实履行职责，扎实工作。今年开始推行政府工作抓落实新机制，把政府全年的主要工作任务分解到各职能部门，建立工作责任制，大兴抓落实、求实效的良好政风。

坚持勤政廉政。以开展“保持共产党员先进性教育活动”为契机，组织“十百千万”干部下基层驻农村、和谐云浮好班子创建活动，使广大公务员树立正确的理想信念和高尚的道德情操，永葆人民公仆本色，出色地履行职责。加强行政监察，完善行政监督制度，对履行职责不力、行政不作为或乱作为的政府部门及相关工作人员要严肃查处；加强审计工作，健全领导干部经济责任、国有企业经营效益和政府投资项目审计制度。今年要开展政府部门的评议活动，以推动机关作风建设，提高部门办事效率和工作质量，努力建设一支“为民、务实、清廉”的公务员队伍。

提高施政能力。继续深入开展普法工作，加强政策法规和社会主义市场经济、法律等知识的学习和运用，努力提高推进全市经济和社会发展全局的能力，抓工作措施落实、完成工作任务的能力，以及解决经济和社会发展中热点难点问题的能力。政府各部门要讲大局、讲风格、讲团结，协调联动，保证政令畅通，努力建设人民满意的政府。

加强民主监督。对全市经济发展战略、重要发展规划、重大建设项目以及关系民生的重要决策，要坚持走群众路线，广泛听取各方面的意见，进行充分论证，提高科学决策水平；认真落实市人大及其常委会的议案，坚持和完善重大事项定期向人大报告、向政协通报制度，认真做好市政府组成人员向人大述职工作。自觉接受人民群众和社会舆论监督，认真办理人民代表的议案、批评、意见、建议和政协委员的提案、建议案，努力把政府工作做得更好。

各位代表，今年是实施“十五”计划的最后一年，也是为“十一五”规划打基础的一年，任务繁重，使命光荣。让我们解放思想，抢抓机遇，团结一致，振奋精神，扎实工作，圆满完成全市经济和社会发展各项任务，加快推进山区经济强市与和谐云浮建设步伐。
